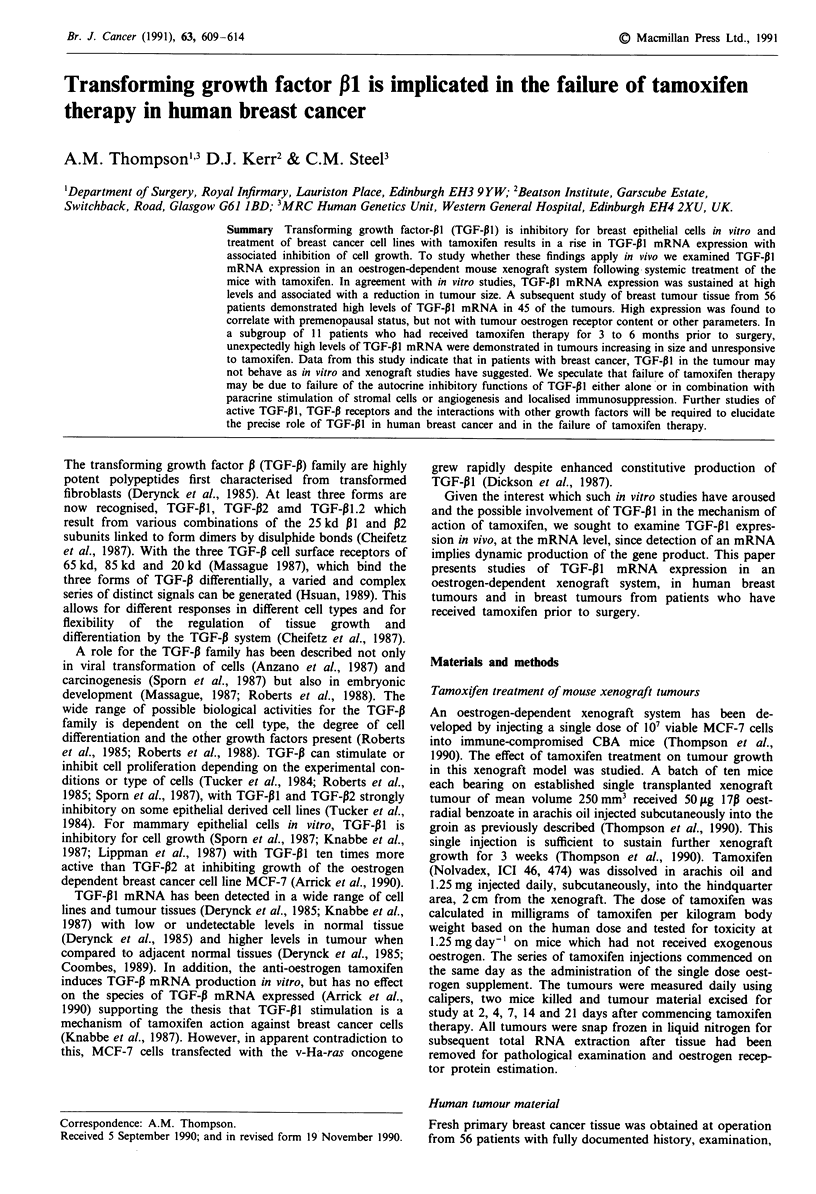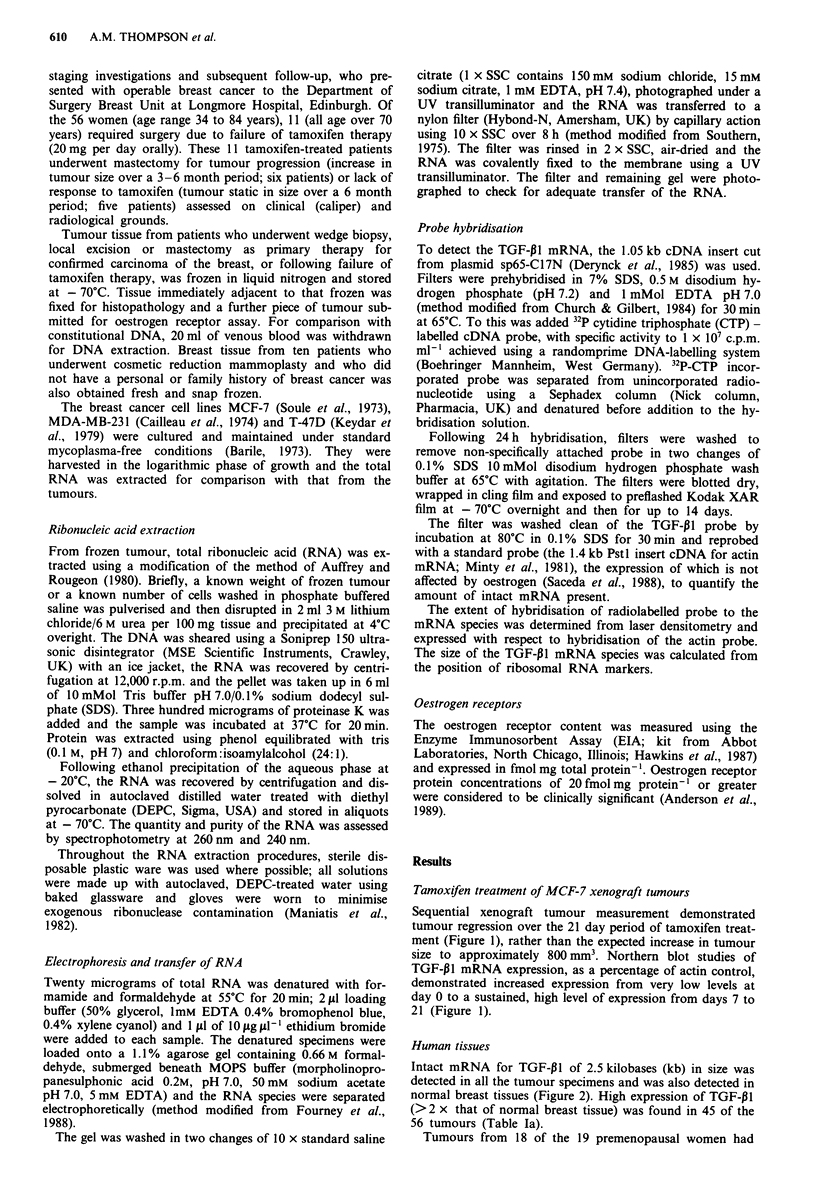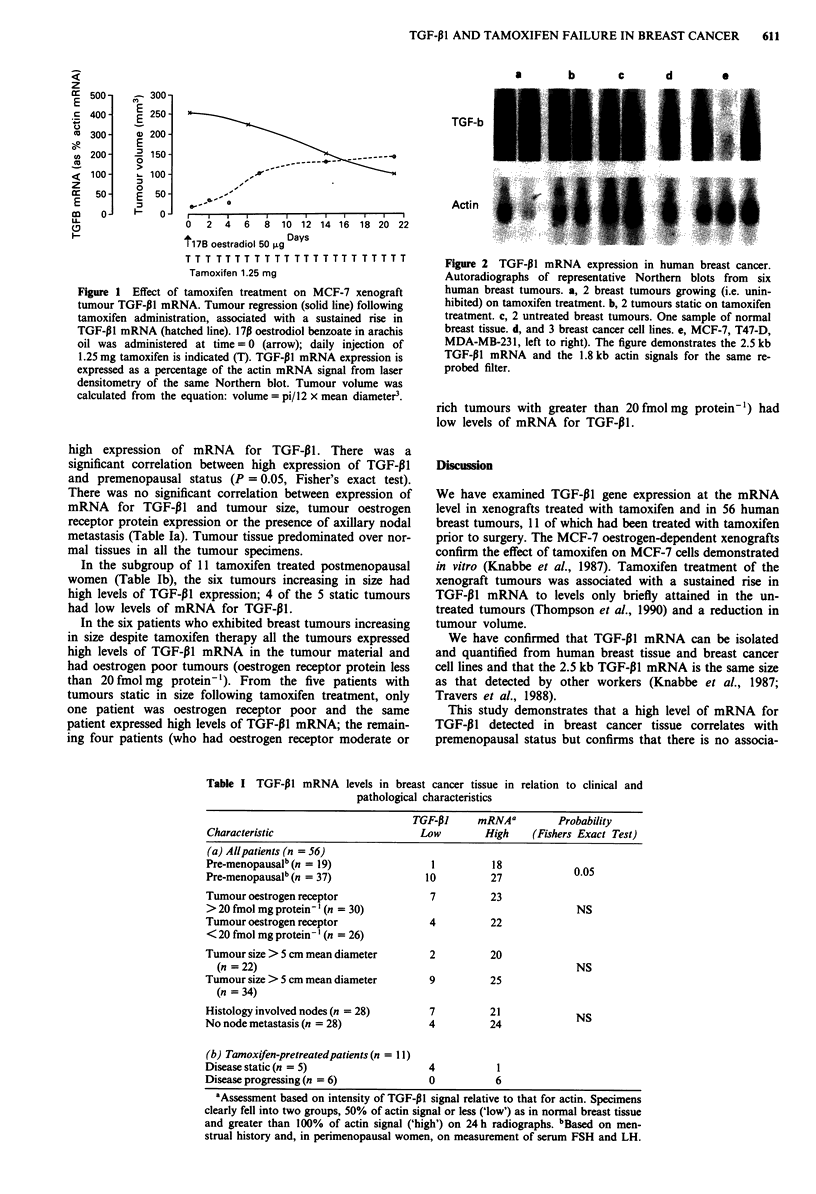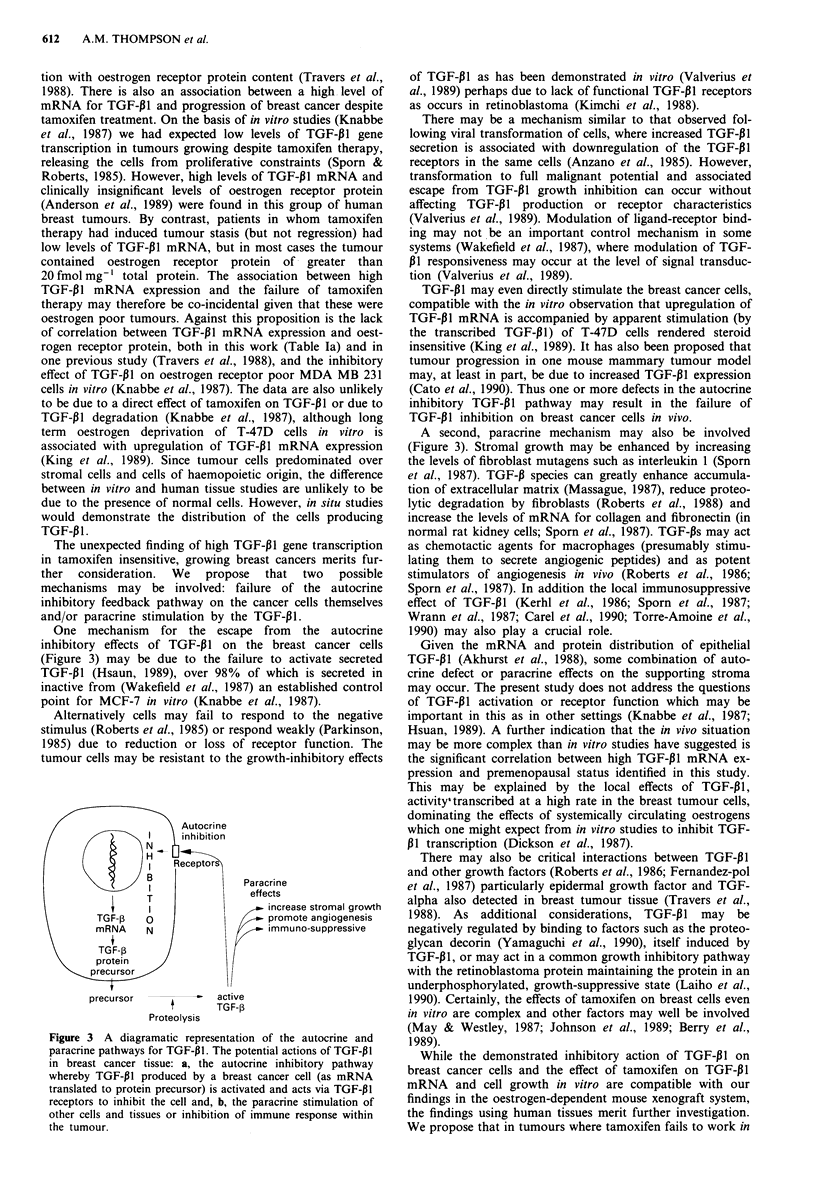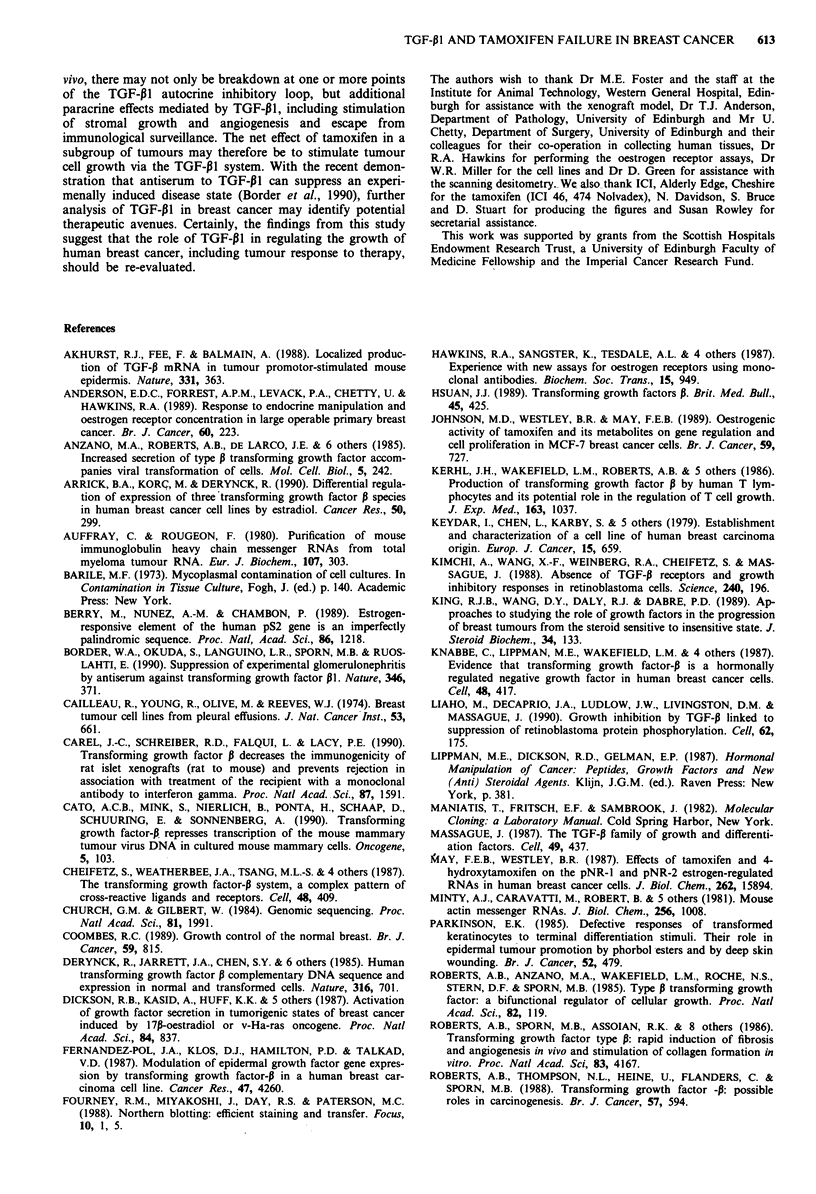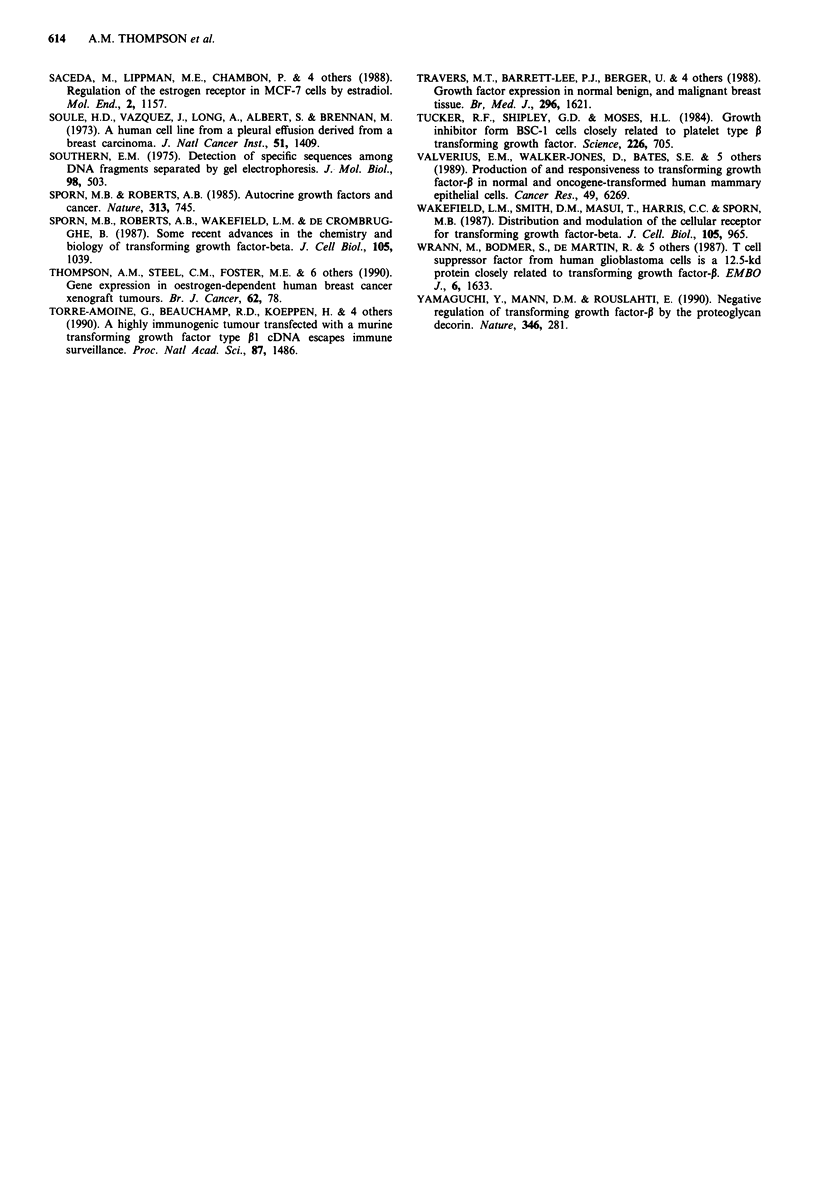# Transforming growth factor beta 1 is implicated in the failure of tamoxifen therapy in human breast cancer.

**DOI:** 10.1038/bjc.1991.140

**Published:** 1991-04

**Authors:** A. M. Thompson, D. J. Kerr, C. M. Steel

**Affiliations:** Department of Surgery, Royal Infirmary, Edinburgh, UK.

## Abstract

**Images:**


Br. J. Cancer (1991), 63, 609-614 © Macmillan Press Ltd., 1991

### Transforming growth factor β1 is implicated in the failure of tamoxifen therapy in human breast cancer

A.M. Thompson[1,3] D.J. Kerr[2] & C.M. Steel[3]

[1]*Department of Surgery, Royal Infirmary, Lauriston Place, Edinburgh EH3 9YW;* [2]*Beatson Institute, Garscube Estate, Switchback, Road, Glasgow G61 1BD;* [3]*MRC Human Genetics Unit, Western General Hospital, Edinburgh EH4 2XU, UK.*

**Summary** Transforming growth factor-β1 (TGF-β1) is inhibitory for breast epithelial cells *in vitro* and treatment of breast cancer cell lines with tamoxifen results in a rise in TGF-β1 mRNA expression with associated inhibition of cell growth. To study whether these findings apply *in vivo* we examined TGF-β1 mRNA expression in an oestrogen-dependent mouse xenograft system following systemic treatment of the mice with tamoxifen. In agreement with *in vitro* studies, TGF-β1 mRNA expression was sustained at high levels and associated with a reduction in tumour size. A subsequent study of breast tumour tissue from 56 patients demonstrated high levels of TGF-β1 mRNA in 45 of the tumours. High expression was found to correlate with premenopausal status, but not with tumour oestrogen receptor content or other parameters. In a subgroup of 11 patients who had received tamoxifen therapy for 3 to 6 months prior to surgery, unexpectedly high levels of TGF-β1 mRNA were demonstrated in tumours increasing in size and unresponsive to tamoxifen. Data from this study indicate that in patients with breast cancer, TGF-β1 in the tumour may not behave as *in vitro* and xenograft studies have suggested. We speculate that failure of tamoxifen therapy may be due to failure of the autocrine inhibitory functions of TGF-β1 either alone or in combination with paracrine stimulation of stromal cells or angiogenesis and localised immunosuppression. Further studies of active TGF-β1, TGF-β receptors and the interactions with other growth factors will be required to elucidate the precise role of TGF-β1 in human breast cancer and in the failure of tamoxifen therapy.

The transforming growth factor β (TGF-β) family are highly potent polypeptides first characterised from transformed fibroblasts (Derynck *et al.*, 1985). At least three forms are now recognised, TGF-β1, TGF-β2 amd TGF-β1.2 which result from various combinations of the 25 kd β1 and β2 subunits linked to form dimers by disulphide bonds (Cheifetz *et al.*, 1987). With the three TGF-β cell surface receptors of 65 kd, 85 kd and 20 kd (Massague 1987), which bind the three forms of TGF-β differentially, a varied and complex series of distinct signals can be generated (Hsuan, 1989). This allows for different responses in different cell types and for flexibility of the regulation of tissue growth and differentiation by the TGF-β system (Cheifetz *et al.*, 1987).

A role for the TGF-β family has been described not only in viral transformation of cells (Anzano *et al.*, 1987) and carcinogenesis (Sporn *et al.*, 1987) but also in embryonic development (Massague, 1987; Roberts *et al.*, 1988). The wide range of possible biological activities for the TGF-β family is dependent on the cell type, the degree of cell differentiation and the other growth factors present (Roberts *et al.*, 1985; Roberts *et al.*, 1988). TGF-β can stimulate or inhibit cell proliferation depending on the experimental conditions or type of cells (Tucker *et al.*, 1984; Roberts *et al.*, 1985; Sporn *et al.*, 1987), with TGF-β1 and TGF-β2 strongly inhibitory on some epithelial derived cell lines (Tucker *et al.*, 1984). For mammary epithelial cells *in vitro*, TGF-β1 is inhibitory for cell growth (Sporn *et al.*, 1987; Knabbe *et al.*, 1987; Lippman *et al.*, 1987) with TGF-β1 ten times more active than TGF-β2 at inhibiting growth of the oestrogen dependent breast cancer cell line MCF-7 (Arrick *et al.*, 1990).

TGF-β1 mRNA has been detected in a wide range of cell lines and tumour tissues (Derynck *et al.*, 1985; Knabbe *et al.*, 1987) with low or undetectable levels in normal tissue (Derynck *et al.*, 1985) and higher levels in tumour when compared to adjacent normal tissues (Derynck *et al.*, 1985; Coombes, 1989). In addition, the anti-oestrogen tamoxifen induces TGF-β mRNA production *in vitro*, but has no effect on the species of TGF-β mRNA expressed (Arrick *et al.*, 1990) supporting the thesis that TGF-β1 stimulation is a mechanism of tamoxifen action against breast cancer cells (Knabbe *et al.*, 1987). However, in apparent contradiction to this, MCF-7 cells transfected with the v-Ha-*ras* oncogene grew rapidly despite enhanced constitutive production of TGF-β1 (Dickson *et al.*, 1987).

Given the interest which such *in vitro* studies have aroused and the possible involvement of TGF-β1 in the mechanism of action of tamoxifen, we sought to examine TGF-β1 expression *in vivo*, at the mRNA level, since detection of an mRNA implies dynamic production of the gene product. This paper presents studies of TGF-β1 mRNA expression in an oestrogen-dependent xenograft system, in human breast tumours and in breast tumours from patients who have received tamoxifen prior to surgery.

### Materials and methods

### *Tamoxifen treatment of mouse xenograft tumours*

An oestrogen-dependent xenograft system has been developed by injecting a single dose of $10^7$ viable MCF-7 cells into immune-compromised CBA mice (Thompson *et al.*, 1990). The effect of tamoxifen treatment on tumour growth in this xenograft model was studied. A batch of ten mice each bearing on established single transplanted xenograft tumour of mean volume 250 mm³ received 50 μg 17β oestradial benzoate in arachis oil injected subcutaneously into the groin as previously described (Thompson *et al.*, 1990). This single injection is sufficient to sustain further xenograft growth for 3 weeks (Thompson *et al.*, 1990). Tamoxifen (Nolvadex, ICI 46, 474) was dissolved in arachis oil and 1.25 mg injected daily, subcutaneously, into the hindquarter area, 2 cm from the xenograft. The dose of tamoxifen was calculated in milligrams of tamoxifen per kilogram body weight based on the human dose and tested for toxicity at 1.25 mg $day^{-1}$ on mice which had not received exogenous oestrogen. The series of tamoxifen injections commenced on the same day as the administration of the single dose oestrogen supplement. The tumours were measured daily using calipers, two mice killed and tumour material excised for study at 2, 4, 7, 14 and 21 days after commencing tamoxifen therapy. All tumours were snap frozen in liquid nitrogen for subsequent total RNA extraction after tissue had been removed for pathological examination and oestrogen receptor protein estimation.

### *Human tumour material*

Fresh primary breast cancer tissue was obtained at operation from 56 patients with fully documented history, examination,

Correspondence: A.M. Thompson.
Received 5 September 1990; and in revised form 19 November 1990.

staging investigations and subsequent follow-up, who presented with operable breast cancer to the Department of Surgery Breast Unit at Longmore Hospital, Edinburgh. Of the 56 women (age range 34 to 84 years), 11 (all age over 70 years) required surgery due to failure of tamoxifen therapy (20 mg per day orally). These 11 tamoxifen-treated patients underwent mastectomy for tumour progression (increase in tumour size over a 3–6 month period; six patients) or lack of response to tamoxifen (tumour static in size over a 6 month period; five patients) assessed on clinical (caliper) and radiological grounds.

Tumour tissue from patients who underwent wedge biopsy, local excision or mastectomy as primary therapy for confirmed carcinoma of the breast, or following failure of tamoxifen therapy, was frozen in liquid nitrogen and stored at − 70°C. Tissue immediately adjacent to that frozen was fixed for histopathology and a further piece of tumour submitted for oestrogen receptor assay. For comparison with constitutional DNA, 20 ml of venous blood was withdrawn for DNA extraction. Breast tissue from ten patients who underwent cosmetic reduction mammoplasty and who did not have a personal or family history of breast cancer was also obtained fresh and snap frozen.

The breast cancer cell lines MCF-7 (Soule *et al.*, 1973), MDA-MB-231 (Cailleau *et al.*, 1974) and T-47D (Keydar *et al.*, 1979) were cultured and maintained under standard mycoplasma-free conditions (Barile, 1973). They were harvested in the logarithmic phase of growth and the total RNA was extracted for comparison with that from the tumours.

*Ribonucleic acid extraction*

From frozen tumour, total ribonucleic acid (RNA) was extracted using a modification of the method of Auffrey and Rougeon (1980). Briefly, a known weight of frozen tumour or a known number of cells washed in phosphate buffered saline was pulverised and then disrupted in 2 ml 3 M lithium chloride/6 M urea per 100 mg tissue and precipitated at 4°C overight. The DNA was sheared using a Soniprep 150 ultrasonic disintegrator (MSE Scientific Instruments, Crawley, UK) with an ice jacket, the RNA was recovered by centrifugation at 12,000 r.p.m. and the pellet was taken up in 6 ml of 10 mMol Tris buffer pH 7.0/0.1% sodium dodecyl sulphate (SDS). Three hundred micrograms of proteinase K was added and the sample was incubated at 37°C for 20 min. Protein was extracted using phenol equilibrated with tris (0.1 M, pH 7) and chloroform:isoamylalcohol (24:1).

Following ethanol precipitation of the aqueous phase at − 20°C, the RNA was recovered by centrifugation and dissolved in autoclaved distilled water treated with diethyl pyrocarbonate (DEPC, Sigma, USA) and stored in aliquots at − 70°C. The quantity and purity of the RNA was assessed by spectrophotometry at 260 nm and 240 nm.

Throughout the RNA extraction procedures, sterile disposable plastic ware was used where possible; all solutions were made up with autoclaved, DEPC-treated water using baked glassware and gloves were worn to minimise exogenous ribonuclease contamination (Maniatis *et al.*, 1982).

*Electrophoresis and transfer of RNA*

Twenty micrograms of total RNA was denatured with formamide and formaldehyde at 55°C for 20 min; 2 $\mu$l loading buffer (50% glycerol, 1mM EDTA 0.4% bromophenol blue, 0.4% xylene cyanol) and 1 $\mu$l of 10 $\mu$g $\mu$l$^{-1}$ ethidium bromide were added to each sample. The denatured specimens were loaded onto a 1.1% agarose gel containing 0.66 M formaldehyde, submerged beneath MOPS buffer (morpholinopropanesulphonic acid 0.2M, pH 7.0, 50 mM sodium acetate pH 7.0, 5 mM EDTA) and the RNA species were separated electrophoretically (method modified from Fourney *et al.*, 1988).

The gel was washed in two changes of 10 × standard saline citrate (1 × SSC contains 150 mM sodium chloride, 15 mM sodium citrate, 1 mM EDTA, pH 7.4), photographed under a UV transilluminator and the RNA was transferred to a nylon filter (Hybond-N, Amersham, UK) by capillary action using 10 × SSC over 8 h (method modified from Southern, 1975). The filter was rinsed in 2 × SSC, air-dried and the RNA was covalently fixed to the membrane using a UV transilluminator. The filter and remaining gel were photographed to check for adequate transfer of the RNA.

*Probe hybridisation*

To detect the TGF-$\beta$1 mRNA, the 1.05 kb cDNA insert cut from plasmid sp65-C17N (Derynck *et al.*, 1985) was used. Filters were prehybridised in 7% SDS, 0.5 M disodium hydrogen phosphate (pH 7.2) and 1 mMol EDTA pH 7.0 (method modified from Church & Gilbert, 1984) for 30 min at 65°C. To this was added $^{32}$P cytidine triphosphate (CTP) – labelled cDNA probe, with specific activity to 1 × 10$^7$ c.p.m. ml$^{-1}$ achieved using a randomprime DNA-labelling system (Boehringer Mannheim, West Germany). $^{32}$P-CTP incorporated probe was separated from unincorporated radionucleotide using a Sephadex column (Nick column, Pharmacia, UK) and denatured before addition to the hybridisation solution.

Following 24 h hybridisation, filters were washed to remove non-specifically attached probe in two changes of 0.1% SDS 10 mMol disodium hydrogen phosphate wash buffer at 65°C with agitation. The filters were blotted dry, wrapped in cling film and exposed to preflashed Kodak XAR film at − 70°C overnight and then for up to 14 days.

The filter was washed clean of the TGF-$\beta$1 probe by incubation at 80°C in 0.1% SDS for 30 min and reprobed with a standard probe (the 1.4 kb Pstl insert cDNA for actin mRNA; Minty *et al.*, 1981), the expression of which is not affected by oestrogen (Saceda *et al.*, 1988), to quantify the amount of intact mRNA present.

The extent of hybridisation of radiolabelled probe to the mRNA species was determined from laser densitometry and expressed with respect to hybridisation of the actin probe. The size of the TGF-$\beta$1 mRNA species was calculated from the position of ribosomal RNA markers.

*Oestrogen receptors*

The oestrogen receptor content was measured using the Enzyme Immunosorbent Assay (EIA; kit from Abbot Laboratories, North Chicago, Illinois; Hawkins *et al.*, 1987) and expressed in fmol mg total protein$^{-1}$. Oestrogen receptor protein concentrations of 20 fmol mg protein$^{-1}$ or greater were considered to be clinically significant (Anderson *et al.*, 1989).

### Results

*Tamoxifen treatment of MCF-7 xenograft tumours*

Sequential xenograft tumour measurement demonstrated tumour regression over the 21 day period of tamoxifen treatment (Figure 1), rather than the expected increase in tumour size to approximately 800 mm$^3$. Northern blot studies of TGF-$\beta$1 mRNA expression, as a percentage of actin control, demonstrated increased expression from very low levels at day 0 to a sustained, high level of expression from days 7 to 21 (Figure 1).

*Human tissues*

Intact mRNA for TGF-$\beta$1 of 2.5 kilobases (kb) in size was detected in all the tumour specimens and was also detected in normal breast tissues (Figure 2). High expression of TGF-$\beta$1 ($>$2 × that of normal breast tissue) was found in 45 of the 56 tumours (Table Ia).

Tumours from 18 of the 19 premenopausal women had

**Figure 1** Effect of tamoxifen treatment on MCF-7 xenograft tumour TGF-β1 mRNA. Tumour regression (solid line) following tamoxifen administration, associated with a sustained rise in TGF-β1 mRNA (hatched line). 17β oestrodiol benzoate in arachis oil was administered at time = 0 (arrow); daily injection of 1.25 mg tamoxifen is indicated (T). TGF-β1 mRNA expression is expressed as a percentage of the actin mRNA signal from laser densitometry of the same Northern blot. Tumour volume was calculated from the equation: volume = pi/12 × mean diameter$^3$.

**Figure 2** TGF-β1 mRNA expression in human breast cancer. Autoradiographs of representative Northern blots from six human breast tumours. **a**, 2 breast tumours growing (i.e. uninhibited) on tamoxifen treatment. **b**, 2 tumours static on tamoxifen treatment. **c**, 2 untreated breast tumours. One sample of normal breast tissue. **d**, and 3 breast cancer cell lines. **e**, MCF-7, T47-D, MDA-MB-231, left to right). The figure demonstrates the 2.5 kb TGF-β1 mRNA and the 1.8 kb actin signals for the same reprobed filter.

high expression of mRNA for TGF-β1. There was a significant correlation between high expression of TGF-β1 and premenopausal status ($P = 0.05$, Fisher's exact test). There was no significant correlation between expression of mRNA for TGF-β1 and tumour size, tumour oestrogen receptor protein expression or the presence of axillary nodal metastasis (Table Ia). Tumour tissue predominated over normal tissues in all the tumour specimens.

In the subgroup of 11 tamoxifen treated postmenopausal women (Table Ib), the six tumours increasing in size had high levels of TGF-β1 expression; 4 of the 5 static tumours had low levels of mRNA for TGF-β1.

In the six patients who exhibited breast tumours increasing in size despite tamoxifen therapy all the tumours expressed high levels of TGF-β1 mRNA in the tumour material and had oestrogen poor tumours (oestrogen receptor protein less than 20 fmol mg protein$^{-1}$). From the five patients with tumours static in size following tamoxifen treatment, only one patient was oestrogen receptor poor and the same patient expressed high levels of TGF-β1 mRNA; the remaining four patients (who had oestrogen receptor moderate or rich tumours with greater than 20 fmol mg protein$^{-1}$) had low levels of mRNA for TGF-β1.

### Discussion

We have examined TGF-β1 gene expression at the mRNA level in xenografts treated with tamoxifen and in 56 human breast tumours, 11 of which had been treated with tamoxifen prior to surgery. The MCF-7 oestrogen-dependent xenografts confirm the effect of tamoxifen on MCF-7 cells demonstrated *in vitro* (Knabbe *et al.*, 1987). Tamoxifen treatment of the xenograft tumours was associated with a sustained rise in TGF-β1 mRNA to levels only briefly attained in the untreated tumours (Thompson *et al.*, 1990) and a reduction in tumour volume.

We have confirmed that TGF-β1 mRNA can be isolated and quantified from human breast tissue and breast cancer cell lines and that the 2.5 kb TGF-β1 mRNA is the same size as that detected by other workers (Knabbe *et al.*, 1987; Travers *et al.*, 1988).

This study demonstrates that a high level of mRNA for TGF-β1 detected in breast cancer tissue correlates with premenopausal status but confirms that there is no associa-

**Table I** TGF-β1 mRNA levels in breast cancer tissue in relation to clinical and pathological characteristics

| *Characteristic* | *TGF-β1 Low* | *mRNA[a] High* | *Probability (Fishers Exact Test)* |
|---|---|---|---|
| *(a) All patients (n = 56)* | | | |
| Pre-menopausal[b] ($n$ = 19) | 1 | 18 | 0.05 |
| Pre-menopausal[b] ($n$ = 37) | 10 | 27 | |
| Tumour oestrogen receptor > 20 fmol mg protein$^{-1}$ ($n$ = 30) | 7 | 23 | NS |
| Tumour oestrogen receptor < 20 fmol mg protein$^{-1}$ ($n$ = 26) | 4 | 22 | |
| Tumour size > 5 cm mean diameter ($n$ = 22) | 2 | 20 | NS |
| Tumour size > 5 cm mean diameter ($n$ = 34) | 9 | 25 | |
| Histology involved nodes ($n$ = 28) | 7 | 21 | NS |
| No node metastasis ($n$ = 28) | 4 | 24 | |
| *(b) Tamoxifen-pretreated patients (n = 11)* | | | |
| Disease static ($n$ = 5) | 4 | 1 | |
| Disease progressing ($n$ = 6) | 0 | 6 | |

[a]Assessment based on intensity of TGF-β1 signal relative to that for actin. Specimens clearly fell into two groups, 50% of actin signal or less ('low') as in normal breast tissue and greater than 100% of actin signal ('high') on 24 h radiographs. [b]Based on menstrual history and, in perimenopausal women, on measurement of serum FSH and LH.

tion with oestrogen receptor protein content (Travers et al., 1988). There is also an association between a high level of mRNA for TGF-β1 and progression of breast cancer despite tamoxifen treatment. On the basis of *in vitro* studies (Knabbe et al., 1987) we had expected low levels of TGF-β1 gene transcription in tumours growing despite tamoxifen therapy, releasing the cells from proliferative constraints (Sporn & Roberts, 1985). However, high levels of TGF-β1 mRNA and clinically insignificant levels of oestrogen receptor protein (Anderson et al., 1989) were found in this group of human breast tumours. By contrast, patients in whom tamoxifen therapy had induced tumour stasis (but not regression) had low levels of TGF-β1 mRNA, but in most cases the tumour contained oestrogen receptor protein of greater than 20 fmol $mg^{-1}$ total protein. The association between high TGF-β1 mRNA expression and the failure of tamoxifen therapy may therefore be co-incidental given that these were oestrogen poor tumours. Against this proposition is the lack of correlation between TGF-β1 mRNA expression and oestrogen receptor protein, both in this work (Table Ia) and in one previous study (Travers et al., 1988), and the inhibitory effect of TGF-β1 on oestrogen receptor poor MDA MB 231 cells *in vitro* (Knabbe et al., 1987). The data are also unlikely to be due to a direct effect of tamoxifen on TGF-β1 or due to TGF-β1 degradation (Knabbe et al., 1987), although long term oestrogen deprivation of T-47D cells *in vitro* is associated with upregulation of TGF-β1 mRNA expression (King et al., 1989). Since tumour cells predominated over stromal cells and cells of haemopoietic origin, the difference between *in vitro* and human tissue studies are unlikely to be due to the presence of normal cells. However, *in situ* studies would demonstrate the distribution of the cells producing TGF-β1.

The unexpected finding of high TGF-β1 gene transcription in tamoxifen insensitive, growing breast cancers merits further consideration. We propose that two possible mechanisms may be involved: failure of the autocrine inhibitory feedback pathway on the cancer cells themselves and/or paracrine stimulation by the TGF-β1.

One mechanism for the escape from the autocrine inhibitory effects of TGF-β1 on the breast cancer cells (Figure 3) may be due to the failure to activate secreted TGF-β1 (Hsaun, 1989), over 98% of which is secreted in inactive from (Wakefield et al., 1987) an established control point for MCF-7 *in vitro* (Knabbe et al., 1987).

Alternatively cells may fail to respond to the negative stimulus (Roberts et al., 1985) or respond weakly (Parkinson, 1985) due to reduction or loss of receptor function. The tumour cells may be resistant to the growth-inhibitory effects

**Figure 3** A diagramatic representation of the autocrine and paracrine pathways for TGF-β1. The potential actions of TGF-β1 in breast cancer tissue: **a**, the autocrine inhibitory pathway whereby TGF-β1 produced by a breast cancer cell (as mRNA translated to protein precursor) is activated and acts via TGF-β1 receptors to inhibit the cell and, **b**, the paracrine stimulation of other cells and tissues or inhibition of immune response within the tumour.

of TGF-β1 as has been demonstrated *in vitro* (Valverius et al., 1989) perhaps due to lack of functional TGF-β1 receptors as occurs in retinoblastoma (Kimchi et al., 1988).

There may be a mechanism similar to that observed following viral transformation of cells, where increased TGF-β1 secretion is associated with downregulation of the TGF-β1 receptors in the same cells (Anzano et al., 1985). However, transformation to full malignant potential and associated escape from TGF-β1 growth inhibition can occur without affecting TGF-β1 production or receptor characteristics (Valverius et al., 1989). Modulation of ligand-receptor binding may not be an important control mechanism in some systems (Wakefield et al., 1987), where modulation of TGF-β1 responsiveness may occur at the level of signal transduction (Valverius et al., 1989).

TGF-β1 may even directly stimulate the breast cancer cells, compatible with the *in vitro* observation that upregulation of TGF-β1 mRNA is accompanied by apparent stimulation (by the transcribed TGF-β1) of T-47D cells rendered steroid insensitive (King et al., 1989). It has also been proposed that tumour progression in one mouse mammary tumour model may, at least in part, be due to increased TGF-β1 expression (Cato et al., 1990). Thus one or more defects in the autocrine inhibitory TGF-β1 pathway may result in the failure of TGF-β1 inhibition on breast cancer cells *in vivo*.

A second, paracrine mechanism may also be involved (Figure 3). Stromal growth may be enhanced by increasing the levels of fibroblast mutagens such as interleukin 1 (Sporn et al., 1987). TGF-β species can greatly enhance accumulation of extracellular matrix (Massague, 1987), reduce proteolytic degradation by fibroblasts (Roberts et al., 1988) and increase the levels of mRNA for collagen and fibronectin (in normal rat kidney cells; Sporn et al., 1987). TGF-βs may act as chemotactic agents for macrophages (presumably stimulating them to secrete angiogenic peptides) and as potent stimulators of angiogenesis *in vivo* (Roberts et al., 1986; Sporn et al., 1987). In addition the local immunosuppressive effect of TGF-β1 (Kerhl et al., 1986; Sporn et al., 1987; Wrann et al., 1987; Carel et al., 1990; Torre-Amoine et al., 1990) may also play a crucial role.

Given the mRNA and protein distribution of epithelial TGF-β1 (Akhurst et al., 1988), some combination of autocrine defect or paracrine effects on the supporting stroma may occur. The present study does not address the questions of TGF-β1 activation or receptor function which may be important in this as in other settings (Knabbe et al., 1987; Hsuan, 1989). A further indication that the *in vivo* situation may be more complex than *in vitro* studies have suggested is the significant correlation between high TGF-β1 mRNA expression and premenopausal status identified in this study. This may be explained by the local effects of TGF-β1, activity' transcribed at a high rate in the breast tumour cells, dominating the effects of systemically circulating oestrogens which one might expect from *in vitro* studies to inhibit TGF-β1 transcription (Dickson et al., 1987).

There may also be critical interactions between TGF-β1 and other growth factors (Roberts et al., 1986; Fernandez-pol et al., 1987) particularly epidermal growth factor and TGF-alpha also detected in breast tumour tissue (Travers et al., 1988). As additional considerations, TGF-β1 may be negatively regulated by binding to factors such as the proteoglycan decorin (Yamaguchi et al., 1990), itself induced by TGF-β1, or may act in a common growth inhibitory pathway with the retinoblastoma protein maintaining the protein in an underphosphorylated, growth-suppressive state (Laiho et al., 1990). Certainly, the effects of tamoxifen on breast cells even *in vitro* are complex and other factors may well be involved (May & Westley, 1987; Johnson et al., 1989; Berry et al., 1989).

While the demonstrated inhibitory action of TGF-β1 on breast cancer cells and the effect of tamoxifen on TGF-β1 mRNA and cell growth *in vitro* are compatible with our findings in the oestrogen-dependent mouse xenograft system, the findings using human tissues merit further investigation. We propose that in tumours where tamoxifen fails to work *in*

*vivo*, there may not only be breakdown at one or more points of the TGF-$\beta$1 autocrine inhibitory loop, but additional paracrine effects mediated by TGF-$\beta$1, including stimulation of stromal growth and angiogenesis and escape from immunological surveillance. The net effect of tamoxifen in a subgroup of tumours may therefore be to stimulate tumour cell growth via the TGF-$\beta$1 system. With the recent demonstration that antiserum to TGF-$\beta$1 can suppress an experimenally induced disease state (Border *et al.*, 1990), further analysis of TGF-$\beta$1 in breast cancer may identify potential therapeutic avenues. Certainly, the findings from this study suggest that the role of TGF-$\beta$1 in regulating the growth of human breast cancer, including tumour response to therapy, should be re-evaluated.

The authors wish to thank Dr M.E. Foster and the staff at the Institute for Animal Technology, Western General Hospital, Edinburgh for assistance with the xenograft model, Dr T.J. Anderson, Department of Pathology, University of Edinburgh and Mr U. Chetty, Department of Surgery, University of Edinburgh and their colleagues for their co-operation in collecting human tissues, Dr R.A. Hawkins for performing the oestrogen receptor assays, Dr W.R. Miller for the cell lines and Dr D. Green for assistance with the scanning desitometry. We also thank ICI, Alderly Edge, Cheshire for the tamoxifen (ICI 46, 474 Nolvadex), N. Davidson, S. Bruce and D. Stuart for producing the figures and Susan Rowley for secretarial assistance.

This work was supported by grants from the Scottish Hospitals Endowment Research Trust, a University of Edinburgh Faculty of Medicine Fellowship and the Imperial Cancer Research Fund.

### References

AKHURST, R.J., FEE, F. & BALMAIN, A. (1988). Localized production of TGF-$\beta$ mRNA in tumour promotor-stimulated mouse epidermis. *Nature*, **331**, 363.

ANDERSON, E.D.C., FORREST, A.P.M., LEVACK, P.A., CHETTY, U. & HAWKINS, R.A. (1989). Response to endocrine manipulation and oestrogen receptor concentration in large operable primary breast cancer. *Br. J. Cancer*, **60**, 223.

ANZANO, M.A., ROBERTS, A.B., DE LARCO, J.E. & 6 others (1985). Increased secretion of type $\beta$ transforming growth factor accompanies viral transformation of cells. *Mol. Cell. Biol.*, **5**, 242.

ARRICK, B.A., KORÇ, M. & DERYNCK, R. (1990). Differential regulation of expression of three transforming growth factor $\beta$ species in human breast cancer cell lines by estradiol. *Cancer Res.*, **50**, 299.

AUFFRAY, C. & ROUGEON, F. (1980). Purification of mouse immunoglobulin heavy chain messenger RNAs from total myeloma tumour RNA. *Eur. J. Biochem.*, **107**, 303.

BARILE, M.F. (1973). Mycoplasmal contamination of cell cultures. In *Contamination in Tissue Culture*, Fogh, J. (ed.) p. 140. Academic Press: New York.

BERRY, M., NUNEZ, A.-M. & CHAMBON, P. (1989). Estrogen-responsive element of the human pS2 gene is an imperfectly palindromic sequence. *Proc. Natl, Acad. Sci.*, **86**, 1218.

BORDER, W.A., OKUDA, S., LANGUINO, L.R., SPORN, M.B. & RUOSLAHTI, E. (1990). Suppression of experimental glomerulonephritis by antiserum against transforming growth factor $\beta$1. *Nature*, **346**, 371.

CAILLEAU, R., YOUNG, R., OLIVE, M. & REEVES, W.J. (1974). Breast tumour cell lines from pleural effusions. *J. Nat. Cancer Inst.*, **53**, 661.

CAREL, J.-C., SCHREIBER, R.D., FALQUI, L. & LACY, P.E. (1990). Transforming growth factor $\beta$ decreases the immunogenicity of rat islet xenografts (rat to mouse) and prevents rejection in association with treatment of the recipient with a monoclonal antibody to interferon gamma. *Proc. Natl Acad. Sci.*, **87**, 1591.

CATO, A.C.B., MINK, S., NIERLICH, B., PONTA, H., SCHAAP, D., SCHUURING, E. & SONNENBERG, A. (1990). Transforming growth factor-$\beta$ represses transcription of the mouse mammary tumour virus DNA in cultured mouse mammary cells. *Oncogene*, **5**, 103.

CHEIFETZ, S., WEATHERBEE, J.A., TSANG, M.L.-S. & 4 others (1987). The transforming growth factor-$\beta$ system, a complex pattern of cross-reactive ligands and receptors. *Cell*, **48**, 409.

CHURCH, G.M. & GILBERT, W. (1984). Genomic sequencing. *Proc. Natl Acad. Sci.*, **81**, 1991.

COOMBES, R.C. (1989). Growth control of the normal breast. *Br. J. Cancer*, **59**, 815.

DERYNCK, R., JARRETT, J.A., CHEN, S.Y. & 6 others (1985). Human transforming growth factor $\beta$ complementary DNA sequence and expression in normal and transformed cells. *Nature*, **316**, 701.

DICKSON, R.B., KASID, A., HUFF, K.K. & 5 others (1987). Activation of growth factor secretion in tumorigenic states of breast cancer induced by 17$\beta$-oestradiol or v-Ha-ras oncogene. *Proc. Natl Acad. Sci.*, **84**, 837.

FERNANDEZ-POL, J.A., KLOS, D.J., HAMILTON, P.D. & TALKAD, V.D. (1987). Modulation of epidermal growth factor gene expression by transforming growth factor-$\beta$ in a human breast carcinoma cell line. *Cancer Res.*, **47**, 4260.

FOURNEY, R.M., MIYAKOSHI, J., DAY, R.S. & PATERSON, M.C. (1988). Northern blotting: efficient staining and transfer. *Focus*, **10**, 1, 5.

HAWKINS, R.A., SANGSTER, K., TESDALE, A.L. & 4 others (1987). Experience with new assays for oestrogen receptors using monoclonal antibodies. *Biochem. Soc. Trans.*, **15**, 949.

HSUAN, J.J. (1989). Transforming growth factors $\beta$. *Brit. Med. Bull.*, **45**, 425.

JOHNSON, M.D., WESTLEY, B.R. & MAY, F.E.B. (1989). Oestrogenic activity of tamoxifen and its metabolites on gene regulation and cell proliferation in MCF-7 breast cancer cells. *Br. J. Cancer*, **59**, 727.

KERHL, J.H., WAKEFIELD, L.M., ROBERTS, A.B. & 5 others (1986). Production of transforming growth factor $\beta$ by human T lymphocytes and its potential role in the regulation of T cell growth. *J. Exp. Med.*, **163**, 1037.

KEYDAR, I., CHEN, L., KARBY, S. & 5 others (1979). Establishment and characterization of a cell line of human breast carcinoma origin. *Europ. J. Cancer*, **15**, 659.

KIMCHI, A., WANG, X.-F., WEINBERG, R.A., CHEIFETZ, S. & MASSAGUE, J. (1988). Absence of TGF-$\beta$ receptors and growth inhibitory responses in retinoblastoma cells. *Science*, **240**, 196.

KING, R.J.B., WANG, D.Y., DALY, R.J. & DABRE, P.D. (1989). Approaches to studying the role of growth factors in the progression of breast tumours from the steroid sensitive to insensitive state. *J. Steroid Biochem.*, **34**, 133.

KNABBE, C., LIPPMAN, M.E., WAKEFIELD, L.M. & 4 others (1987). Evidence that transforming growth factor-$\beta$ is a hormonally regulated negative growth factor in human breast cancer cells. *Cell*, **48**, 417.

LIAHO, M., DECAPRIO, J.A., LUDLOW, J.W., LIVINGSTON, D.M. & MASSAGUE, J. (1990). Growth inhibition by TGF-$\beta$ linked to suppression of retinoblastoma protein phosphorylation. *Cell*, **62**, 175.

LIPPMAN, M.E., DICKSON, R.D., GELMAN, E.P. (1987). *Hormonal Manipulation of Cancer: Peptides, Growth Factors and New (Anti) Steroidal Agents.* Klijn, J.G.M. (ed.). Raven Press: New York, p. 381.

MANIATIS, T., FRITSCH, E.F. & SAMBROOK, J. (1982). *Molecular Cloning: a Laboratory Manual.* Cold Spring Harbor, New York.

MASSAGUE, J. (1987). The TGF-$\beta$ family of growth and differentiation factors. *Cell*, **49**, 437.

MAY, F.E.B., WESTLEY, B.R. (1987). Effects of tamoxifen and 4-hydroxytamoxifen on the pNR-1 and pNR-2 estrogen-regulated RNAs in human breast cancer cells. *J. Biol. Chem.*, **262**, 15894.

MINTY, A.J., CARAVATTI, M., ROBERT, B. & 5 others (1981). Mouse actin messenger RNAs. *J. Biol. Chem.*, **256**, 1008.

PARKINSON, E.K. (1985). Defective responses of transformed keratinocytes to terminal differentiation stimuli. Their role in epidermal tumour promotion by phorbol esters and by deep skin wounding. *Br. J. Cancer*, **52**, 479.

ROBERTS, A.B., ANZANO, M.A., WAKEFIELD, L.M., ROCHE, N.S., STERN, D.F. & SPORN, M.B. (1985). Type $\beta$ transforming growth factor: a bifunctional regulator of cellular growth. *Proc. Natl Acad. Sci.*, **82**, 119.

ROBERTS, A.B., SPORN, M.B., ASSOIAN, R.K. & 8 others (1986). Transforming growth factor type $\beta$: rapid induction of fibrosis and angiogenesis *in vivo* and stimulation of collagen formation *in vitro*. *Proc. Natl Acad. Sci*, **83**, 4167.

ROBERTS, A.B., THOMPSON, N.L., HEINE, U., FLANDERS, C. & SPORN, M.B. (1988). Transforming growth factor -$\beta$: possible roles in carcinogenesis. *Br. J. Cancer*, **57**, 594.

SACEDA, M., LIPPMAN, M.E., CHAMBON, P. & 4 others (1988). Regulation of the estrogen receptor in MCF-7 cells by estradiol. *Mol. End.*, **2,** 1157.

SOULE, H.D., VAZQUEZ, J., LONG, A., ALBERT, S. & BRENNAN, M. (1973). A human cell line from a pleural effusion derived from a breast carcinoma. *J. Natl Cancer Inst.*, **51,** 1409.

SOUTHERN, E.M. (1975). Detection of specific sequences among DNA fragments separated by gel electrophoresis. *J. Mol. Biol.*, **98,** 503.

SPORN, M.B. & ROBERTS, A.B. (1985). Autocrine growth factors and cancer. *Nature*, **313,** 745.

SPORN, M.B., ROBERTS, A.B., WAKEFIELD, L.M. & DE CROMBRUGGHE, B. (1987). Some recent advances in the chemistry and biology of transforming growth factor-beta. *J. Cell Biol.*, **105,** 1039.

THOMPSON, A.M., STEEL, C.M., FOSTER, M.E. & 6 others (1990). Gene expression in oestrogen-dependent human breast cancer xenograft tumours. *Br. J. Cancer*, **62,** 78.

TORRE-AMOINE, G., BEAUCHAMP, R.D., KOEPPEN, H. & 4 others (1990). A highly immunogenic tumour transfected with a murine transforming growth factor type β1 cDNA escapes immune surveillance. *Proc. Natl Acad. Sci.*, **87,** 1486.

TRAVERS, M.T., BARRETT-LEE, P.J., BERGER, U. & 4 others (1988). Growth factor expression in normal benign, and malignant breast tissue. *Br, Med. J.*, **296,** 1621.

TUCKER, R.F., SHIPLEY, G.D. & MOSES, H.L. (1984). Growth inhibitor form BSC-1 cells closely related to platelet type β transforming growth factor. *Science*, **226,** 705.

VALVERIUS, E.M., WALKER-JONES, D., BATES, S.E. & 5 others (1989). Production of and responsiveness to transforming growth factor-β in normal and oncogene-transformed human mammary epithelial cells. *Cancer Res.*, 49, 6269.

WAKEFIELD, L.M., SMITH, D.M., MASUI, T., HARRIS, C.C. & SPORN, M.B. (1987). Distribution and modulation of the cellular receptor for transforming growth factor-beta. *J. Cell. Biol.*, **105,** 965.

WRANN, M., BODMER, S., DE MARTIN, R. & 5 others (1987). T cell suppressor factor from human glioblastoma cells is a 12.5-kd protein closely related to transforming growth factor-β. *EMBO J.*, **6,** 1633.

YAMAGUCHI, Y., MANN, D.M. & ROUSLAHTI, E. (1990). Negative regulation of transforming growth factor-β by the proteoglycan decorin. *Nature*, **346,** 281.